# Immunotherapy in hematologic malignancies: past, present, and future

**DOI:** 10.1186/s13045-017-0453-8

**Published:** 2017-04-24

**Authors:** Annie Im, Steven Z. Pavletic

**Affiliations:** 1 University of Pittsburgh Cancer Institute, 5150 Centre Ave, Suite 554, Pittsburgh, PA 15213 USA; 2 National Cancer Institute, National Institutes of Health, Bethesda, MD USA

**Keywords:** Immunotherapy, Hematologic malignancies, Stem cell transplantation

## Abstract

The field of immunotherapy in cancer treatments has been accelerating over recent years and has entered the forefront as a leading area of ongoing research and promising therapies that have changed the treatment landscape for a variety of solid malignancies. Prior to its designation as the Science Breakthrough of the Year in 2013, cancer immunotherapy was active in the treatment of hematologic malignancies. This review provides a broad overview of the past, present, and potential future of immunotherapy in hematologic malignancies.

## Background

The field of immunotherapy in cancer treatments has been accelerating over recent years and has entered the forefront as a leading area of ongoing research and promising therapies that have changed the treatment landscape for a variety of solid malignancies. Prior to its designation as the Science Breakthrough of the Year in 2013 [1], cancer immunotherapy was active in the treatment of hematologic malignancies. This review provides a broad overview of the past, present, and potential future of immunotherapy in hematologic malignancies.

## The past (and present)

Those in the field of treating hematologic malignancies can boast utilizing one of the oldest forms of cancer immunotherapy: allogeneic hematopoietic stem cell transplantation (HCT). The first allogeneic transplant was performed in 1968 by E. Donnall Thomas, who would go on to win the Nobel Prize for being a pioneer in this technology and the father of stem cell transplantation. Although it has been referred to as “the chemotherapist’s bluntest weapon” [2], as it indeed aims to eradicate the hematopoietic and immune systems of a patient, it is often the only curative option for many patients with hematologic malignancies. Moreover, with widening clinical indications and the use of alternative donors, its use only continues to increase [3]. In addition, allogeneic HCT has provided a model of immunotherapy in hematologic malignancies, offering invaluable information that can be used as the field moves forward. For example, we have learned about the sensitivity of hematologic malignancies to the “graft-versus-leukemia” or “graft-versus-tumor” effect, and thus, we know these are appropriate targets for immunotherapy [4, 5]. This sensitivity has been demonstrated by several factors: (1) the efficacy of allogeneic transplant in chemo-refractory disease [6, 7], (2) the use of donor lymphocyte infusions to treat relapse after transplant [8, 9], and (3) the use of reduced-intensity or non-myeloablative conditioning regimens (“mini-transplant”) [10], where the reliance of efficacy and disease eradication is primarily on the graft-versus-leukemia effect, with little contribution from high-dose chemotherapy.

The overall survival after allogeneic transplant has improved significantly over the past several decades [11], although this is mainly due to improvements in non-relapse mortality and advances in supportive care (treatment and prevention of infections or graft-versus-host disease (GVHD), for example) [12]. In addition, novel transplant strategies have been refined to overcome obstacles such as donor availability. For example, haploidentical donor transplant using post-transplant cyclophosphamide as GVHD prophylaxis has shown outcomes similar to matched unrelated donor transplants with lower risk of GVHD [13, 14]. Strategies such as this have widened the applicability of allogeneic transplant and will impact the field of transplantation moving forward. Unfortunately, relapse of the underlying malignancy remains the most common cause of failure or death after transplant, underscoring the need to improve the way in which we are able to harness the immune system to treat hematologic malignancies and how far we still have to go to achieve cure. Nonetheless, we have learned, and continue to learn, immensely about the immune system in the treatment of hematologic malignancies from the evolving and advancing field of allogeneic HCT, which has helped to move the field of novel immunotherapies forward.

### Novel aspects of hematologic malignancies

Although immunotherapy has shown success in a wide variety of cancers including many solid tumors, there are some unique features of hematologic malignancies in this setting that makes these cancers well poised as targets of immunotherapy [15]. First is the sensitivity to immune attack, as previously discussed. In addition, cells of the immune system and cells of the malignancy are in constant contact with each other within the hematopoietic system, making the environment conducive to constant immune surveillance. Moreover, since the cellular origins of the malignancy are those of the immune system, these malignant cells are immunostimulatory by their nature. Finally, for the purposes of research and being able to study the immune mechanisms in these malignancies, these malignancies are accessible and thus easy to sample, especially before and after treatment.

In contrast to these advantages, there are also some disadvantages that hematologic malignancies carry with them in this setting, related to these same factors. As mentioned, the cellular origins are the same between the malignancy and the immune system. Although this is in some ways advantageous, the disadvantage is that the malignant cells themselves may also be stimulated by the inflammatory response and cytokine milieu. Also, given that these cells are by their very nature exploitations of the normal immune system, we know that the normal immune response is at a deficit and may be hindered overall [16]. Finally, and importantly, the mechanisms by which the malignancies have achieved immune evasion are likely exceptional, given the close contact with normal immune cells as previously mentioned [17]. Thus, successful immune strategies have to be able to overcome these immune escape mechanisms.

This review will focus on five categories of immunotherapies in the treatment of hematologic malignancies in which there have been accelerating development, based on various strategies of harnessing the immune system. It should be noted that there is ongoing research and development of other novel strategies that may be entering the clinical arena in the near future.

## The present

### Monoclonal antibodies

Rituximab, an anti-CD20 monoclonal antibody targeting B cells, was the first monoclonal antibody to be approved by the United States Food and Drug Administration (FDA) for the treatment of cancer in 1997 and since has become the prototype for anti-CD20 monoclonal antibodies and a backbone of B cell malignancy treatment regimens. It is a type I antibody and thus exhibits complement-dependent cytotoxicity and antibody-dependent cytotoxicity. Since rituximab, newer monoclonal antibodies directed against CD20 have been developed for use in B cell malignancies, including ofatumumab and obinutuzumab. Ofatumumab is a second generation, fully humanized anti-CD20 monoclonal antibody that binds to a different site than rituximab, and is also a type I antibody. This agent was FDA approved for the treatment of previously treated chronic lymphocytic leukemia (CLL) in 2009 or in combination with chlorambucil for the treatment of previously untreated CLL in 2014 [18, 19]. Obinutuzumab is another second-generation anti-CD20 monoclonal antibody that is a glycoengineered type II antibody, which differs from the others in that it induces direct cell death, along with enhanced antibody-dependent cytotoxicity. This agent was FDA approved in combination with chlorambucil for the treatment of previously untreated CLL in 2013 and in combination with bendamustine for the treatment of relapsed/refractory follicular lymphoma (FL) in 2016 [20, 21]. Studies are ongoing with both of these agents, which may highlight the best role of these agents in the context of current B cell malignancy regimens.

Multiple myeloma (MM) had been long left out of the monoclonal antibody arena until recently, with the surge in development of effective monoclonal antibody therapies based on identification of target antigens. Two of these agents have been FDA approved in the treatment of relapsed/refractory multiple myeloma and show much promise. Daratumumab is an anti-CD38 monoclonal antibody that was FDA approved for the treatment of MM in patients who received at least three prior therapies including a proteasome inhibitor and an immunomodulatory agent. CD38 is a transmembrane glycoprotein that is ubiquitously expressed on MM cells, and other monoclonal antibodies targeting this antigen are in development. Daratumumab induces overall response rates (ORR) of 29–36% in heavily pretreated patients as a single agent [22–24]. In two recent randomized controlled trials, there was significantly improved ORR of 83–93% when daratumumab was added to bortezomib or lenalidomide and dexamethasone, compared to these agents alone, with 61–63% reduction in risk of progression or death [25, 26]. Elotuzumab is an anti-SLAMF7 (or CS1) monoclonal antibody that was FDA approved in combination with lenalidomide and dexamethasone for the treatment of relapsed/refractory MM in November 2015. SLAMF7 is a glycoprotein that is expressed on both MM cells and natural killer cells and exhibits antitumor effects through antibody-dependent cytotoxicity, as well as enhancing activation of NK cell cytotoxicity via SLAMF7 ligation, thus having dual targets. It has shown significantly improved 1-year and 2-year progression-free survival (PFS) of 68 and 41% compared in combination with lenalidomide and dexamethasone compared to these agents alone, with an ORR of 79% [27]. Of note, aside from infusion reactions, the addition of both of these monoclonal antibodies to current MM treatment regimens has not led to additive toxicities. Other combination trials are ongoing evaluating different combination regimens and clinical settings for these and other monoclonal antibodies in MM. In addition to CD38 and SLAMF7, other potential targets include CD138, CD56, CD40, and B cell-activating factor (BAFF) [28]. These agents are likely to change the treatment paradigms of MM in the near future.

### Antibody-drug conjugates

Antibody-drug conjugates take monoclonal antibodies a step further by linking the targeting antibody with a cytotoxic agent. The initial excitement of these direct drug delivery systems rose and fell with the approval and subsequent withdrawal of gemtuzumab ozogamicin (GO) in 2000 and 2010, respectively. However, a recent meta-analysis from five randomized controlled trials that included 3325 patients with acute myeloid leukemia (AML) demonstrated that the use of GO improved 5-year overall survival and reduced the risk of relapse [29]. The absolute survival benefit was especially apparent in patients with favorable and intermediate risk cytogenetics. These data among others have prompted reconsideration of the withdrawal of GO. Nonetheless, recent momentum has been gained in hematologic malignancies again with the development of brentuximab, an anti-CD30 antibody linked to a microtubule inhibitor, MMAE. Because of its efficacy in the treatment of relapsed/refractory Hodgkin lymphoma (HL) (either after autologous stem cell transplant or in patients who are not transplant candidates) and in relapsed systemic anaplastic large cell lymphoma, which are both malignancies in which CD30 is highly expressed, brentuximab received FDA approval for the treatment of both of these diseases in 2011 [30, 31]. It also received approval for post-autologous stem cell transplant consolidation in patients with HL at high risk of relapse or progression in 2015, based on data showing a median PFS of 43 months compared to 24 months for patients who received placebo [32].

Inotuzumab ozogamicin (IO) is another antibody-drug conjugate that has been tested in hematologic malignancies. This agent targets CD22 and is linked to the potent antitumor antibiotic calicheamicin. IO was recently shown to have significantly improved complete remission (CR) rate of 81% compared to standard therapy in a phase 3 trial in patients with relapsed or refractory acute lymphoblastic leukemia (ALL) [33], a patient population in whom outcomes would otherwise be dismal. This agent may dramatically impact the outcomes of patients with ALL as further study is ongoing. Another antibody-drug conjugate that has recently gained attention is SGN-CD33A, which links an anti-CD33 antibody, targeting AML cells, with a pyrrolobenzodiazapine dimer. CD33 is expressed on cells with myeloid lineage and was the target for the antibody in GO. SGN-CD33A has shown a CR rate of 33% in relapsed AML as monotherapy and CR rates of 60 and 65% as monotherapy or in combination with a hypomethylating agent in unfit AML, respectively [34, 35]. Importantly, it has not displayed any signal of concerning hepatotoxicity, which was associated with GO. Other targets for antibody-drug conjugates that are in development include CD138, CD19, and CD33.

### Bispecific T cell engagers

Another exciting area of novel immunotherapies are the bispecific T cell engagers (BiTEs). These agents have two antibody variable fragments, one that includes anti-CD3, that are joined by a linker, and thus have dual specificity for CD3 on T cells as well as a tumor surface antigen [36, 37]. These agents physically bring together T cells and the tumor cells to catalyze the formation of the immunologic synapse and lead to a polyclonal T cell response and cytotoxicity of the tumor cell. Importantly, this process is independent of MHC expression, thus bypassing one of the mechanisms of tumor immune evasion. CD19 is a marker on most B cell malignancies and is specific to B cells, making it an ideal target in immunotherapy for these cancers, which will be highlighted in BiTEs and in the discussion of chimeric antigen receptor (CAR) T cells.

The prototype for BiTEs is blinatumomab, which has dual specificity for CD3 and CD19, and has been shown to be effective in patients with relapsed or refractory B cell ALL [38, 39]. In a phase 2 study in patients with relapsed/refractory Philadelphia chromosome (Ph)-negative B cell ALL, the CR/CR with incomplete count recovery (CRi) rate was 43%, and half of these patients were able to proceed to allogeneic stem cell transplantation [40]. An earlier study demonstrated a CR/CRi rate of 69%, 28% of whom went on to have overall survival (OS) ≥30 months [41, 42]. Based on these data, blinatumomab received accelerated FDA approval for the treatment of relapsed or refractory Ph-negative B cell ALL in December 2014. It has also been studied in the setting of Ph-positive B cell ALL after treatment with tyrosine-kinase inhibitor therapy showing a CR/CRi rate of 36%, including those with a T315I mutation, half of whom went on to undergo allogeneic stem cell transplantation, and has also been used in patients with minimal residual disease (MRD) after chemotherapy to achieve MRD-negative disease, leading to improved survival [43, 44]. Studies in relapsed/refractory non-Hodgkin lymphoma (NHL) including diffuse large B cell lymphoma have been promising, showing overall response rates of 43–69% [45, 46]. The primary concerning toxicities are the cytokine release syndrome (CRS) and neurotoxicity, which will be discussed further in the discussion of CAR T cell therapies. In addition, this agent requires continuous intravenous administration for 4 weeks of a 6-week cycle, which may be a feasibility issue, but can be done in the outpatient setting. Additional targets that are being developed include CD33 BiTEs for AML and also bispecific NK cell engagers (“BiKEs”) linking CD16 with a tumor target antigen.

### CAR T cells

An incredibly promising area for immunotherapy in hematologic malignancies has been the development and refinement of CAR T cell therapy, which is a field that is moving at an accelerated pace. This therapy involves not only targeting tumor antigens directly but also augmentation of these targeted immune effectors. CAR T cells are autologous T cells that are engineered to express chimeric antigen receptors against a specific tumor surface antigen, thus are antigen specific *and* HLA independent, and therefore are independent of MHC expression. The general anatomy of CARs includes a single-chain variable fragment derived from an antibody, linked by a hinge and transmembrane domain to an intracellular T cell signaling domain with a costimulatory domain (number and type depending on the specific CAR) [47, 48]. This strategy has been particularly successful in hematologic malignancies, given several advantages compared to solid tumors: there are established cell surface antigens to target (e.g., CD19 on B cell malignancies); tumor sampling is straightforward and less invasive than in solid tumors, as discussed previously; and importantly, there is already a natural homing of T cells to the areas where the malignancy is located, e.g., blood, bone marrow, lymph nodes, thus making the road for these CARs straightforward.

The process by which patients undergo CAR T cell therapy begins with collection of autologous T cells by leukapheresis. The CAR is then introduced into the T cells through one of the several mechanisms, most commonly using viral vectors, and then, the cells undergo culture for expansion. Usually, patients undergo lymphodepleting chemotherapy prior to CAR T cell infusion, which can enhance in vivo expansion of T cells through the expression of homeostatic cytokines, such as IL-7 and IL-15. This in vivo expansion has been correlated with response to therapy, thus may be more significant than the actual dose of T cells that are infused [49–52]. The engagement of tumor antigen by CAR to the T cells then leads to cytotoxicity and massive T cell proliferation, which again is going to be independent of MHC expression.

The first successful CAR T cell therapies have targeted CD19 in B cell malignancies. Table 1 summarizes published clinical trials using CD19 CAR T cells, which are from only a few institutions across the country. These studies have been performed in patients with NHL, CLL, and ALL, all with relapsed and/or chemo-refractory disease. With the exception of the first study, which utilized a first-generation CAR T cell and yielded no responses, all of these trials used second-generation CAR T cells, which are defined as CARs that include the single costimulatory domain derived from either CD28 or 4-1BB. Third-generation CARs include two costimulatory domains and are currently being evaluated in clinical trials. Table 1 highlights the response rates with CAR T cell therapies in heavily pretreated, relapsed, and refractory patient populations. In particular, responses in ALL have been extremely successful in patients who would otherwise have dismal outcomes. There are also studies showing responses in patients with relapsed disease after allogeneic stem cell transplant, another clinical setting in which salvage is rare, by using CAR T cells from the stem cell donor. Of note, even with responses, there was no evidence of GVHD in these studies. All of these trials have also demonstrated in vivo duration of the CAR T cells, which is associated with duration of response, and the observation that responses were generally correlated with the presence of the cytokine release syndrome, a potentially fatal consequence of CAR T cell therapy.

**Table 1:** Clinical trials of CD19 CAR T cells

Study	Institution	No. of patients	Disease (all relapsed/refractory)	Outcomes	Duration of CARs	Best duration of response	Comments
Jensen et al. (2010) [74]	City of Hope	4	NHL	No responses (2 with CR after autoHCT)	1 week	–	1st generation CAR
Kochenderfer et al., (2010, 2012) [75, 76]	NCI	8	NHL (4) and CLL (4)	80% ORR (1 CR, 5 PR)	Up to 6 months	>18 months	5/8 CRS 4/8 B cell aplasia
Savoldo et al. (2011) [77]	Baylor	6	NHL	2 SD	2nd generation—up to 6 months	–	Infusion of 1st and 2nd generation T cells in the same patient
Brentjens et al. (2011) [78]	MSKCC	10	CLL (8) and ALL (2)	CLL—1 PR, 2 SD ALL—1 durable B cell aplasia	Up to 6 weeks (correlated with burden of disease)	8 months	Most with CRS
Porter et al. and Kalos et al. (2011) [79, 80]	UPenn	3	CLL	2 CR, 1PR	Up to 6 months	>11 months	All with CRS
Brentjens et al., and Davila et al. (2013, 2014) [54, 81]	MSKCC	16	ALL	88% CR (78% in refractory disease)	Up to 4 months	3 months (but 7 went on to alloHCT)	7 CRS CRP, IFNγ, IL6 correlated with CRS
Kochenderfer et al. (2013) [82]	NCI	10	CLL (4) and NHL (6) post alloHCT	1 PR, 1 CR, 6 SD	Up to 30 days (used donor T cells)	9 months	No GVHD
Kochenderfer et al. (2014) [83]	NCI	15	NHL (DLBCL and indolent lymphomas)	8 CR, 4 PR, 1 SD	Up to 11 weeks	22 months	6/7 DLBCL with response
Lee et al. (2014) [49]	NCI	21	ALL (20), NHL (1), 8 post alloHCT	14 CR (13 MRD negative), correlated with CAR expansion	Up to 8 weeks (most went on to alloHCT)	19 months	3 severe CRS CRP, IL6, and CAR expansion correlated with CRS
Grupp et al. and Maude et al. (2013, 2014) [52, 84]	UPenn	30	ALL, 18 post alloHCT	90% CR (15 post alloHCT, 2 post blinatumomab)	Up to 2 years	24 months	All with CRS
Porter et al. (2015) [53]	UPenn	14	CLL	57% ORR (4 CR and 4 PR)	>4 years (no relapses)	>4 years	All with CRS
Brudno et al. (2016) [50]	NCI	20	B cell malignancies post alloHCT	6 CR, 2 PR (4/5 ALL patients achieved MRD negativity)	–	>30 months (CR)	No new-onset GVHD; Higher peak blood CAR T cell levels correlated with response
Turtle et al. (2016) [85]	FHCRC	29	ALL (12 post alloHCT)	93% CR (27/29), 25 with MRD negativity	–	–	25 CRS (3 deaths); No GVHD in post-alloHCT patients; CAR T cell grafts with defined CD4:CD8 ratio

*NHL* non-Hodgkin’s lymphoma, *CLL* chronic lymphocytic leukemia, *ALL* acute lymphoblastic leukemia, *autoHCT* autologous hematopoietic stem cell transplant, *NCI* National Cancer Institute, *ORR* overall response rate, *CR* complete response, *PR* partial response, *SD* stable disease, *CRS* cytokine release syndrome, *MSKCC* Memorial Sloan Kettering Cancer Center, *UPenn* University of Pennsylvania, *alloHCT* allogeneic hematopoietic stem cell transplant, *CRP* C-reactive protein, *IFNγ* interferon-γ, *IL6* interleukin-6, *GVHD* graft-versus-host disease, *DLBCL* diffuse large B cell lymphoma, *MRD* minimal residual disease, *FHCRC* Fred Hutchinson Cancer Research Center

From these early experiences with CAR T cells, there have been some important lessons that have been learned. For example, durable remissions are possible in relapsed/refractory NHL, CLL, and ALL, and the persistence of circulating CAR T cells has been seen more than 4 years after infusion in patients with CLL [53]. In addition, remarkable CR rates of 90% have been seen in relapsed/refractory ALL, which is significant compared to historical controls [52, 54]. Moreover, CAR T cells have been effective in pre- and post-transplant disease settings and chemo-refractory disease, areas where standard therapies have typically failed. Interestingly, central nervous system disease has been cleared with CAR T cell therapy as well, an area that standard therapies do not penetrate. Also, although factors that are predictive for response are still being studied, response does seem to correlate with the in vivo expansion of CAR T cells (rather than the infused dose) and the presence of the cytokine release syndrome. Finally, when relapses occur, antigen-positive relapses tend to occur after CAR T cells are no longer in circulation. Interestingly, B cell aplasia, an on-target, off-tumor effect, can be a surrogate for the persistence of CAR T cells. When antigen-negative relapses occur, this may happen despite the presence of circulating CAR T cells, and strategies are being developed to try to overcome this, such as dual antigen target CAR T cells. Based on the exciting early experiences with CAR T cell therapy, CTL019, a CD19 CAR T cell construct owned by Novartis, received FDA breakthrough therapy designation in July 2014 for the treatment of relapsed/refractory ALL. There is much ongoing work in antigen discovery for other malignancies, such as B cell maturation antigen (BCMA) in multiple myeloma [8], or CD123 in AML [55].

A discussion about CAR T cells would not be complete without highlighting the CRS, an inflammatory process related to exponential T cell proliferation associated with massive cytokine elevation. Important lessons learned from early experiences with CAR T cells have demonstrated the necessity of this response and the appropriate management of the clinical syndrome. Although the presence of CRS may correlate with response, the severity does not seem to be related to response, suggesting that efforts to decrease the severity of the syndrome are appropriate. Nonetheless, it is an expected manifestation of CAR T cell therapy and potentially fatal if not managed appropriately; thus, guidelines have been developed for recommended diagnosis and management of CRS [56]. Clinically, CRS is characterized by very high fevers and flu-like symptoms and, when severe, can lead to vascular leak, hypotension and hemodynamic instability, and multi-organ failure. Management of CRS not uncommonly necessitates transfer to the intensive care unit. The only known predictor of CRS at this time is disease burden at the time of transfer; however, there have also been correlations with levels of C-reactive protein (CRP) and IL-6 prior to the development of CRS that have been demonstrated [49]. An agent that has become important in the management of CRS is tocilizumab, an anti-IL-6 agent that is approved for the treatment of rheumatoid arthritis. Because of the rise in IL-6 associated with CRS, this agent was used in early experiences and was found to be extremely effective at treating even life-threatening CRS, while not impacting the antitumor response. Although steroids have been used in the management of CRS, there is theoretical concern about dampening the T cell response and impacting efficacy. As previously mentioned, CRS is seen with BiTE therapy as well, and tocilizumab is used in the management of CRS in this clinical setting as well.

Other toxicities that are associated with CAR T cell therapy include neurotoxicity, which can include headaches, confusion, hallucinations, dysphasia, ataxia, apraxia, facial nerve palsy, tremor, dysmetria, global encephalopathy, and even seizures [57]. The reported incidence varies between 0 and 50%. This is an acute toxicity that is not related to CRS, as it has developed after the occurrence and treatment of CRS, and is not prevented by prior treatment with tocilizumab. However, most cases resolve on their own and are self-limited without any known long-term or persistent deficits [58]. In terms of chronic toxicities, the on-target, off-tumor effect of B cell aplasia was already mentioned as a surrogate for the persistence of CAR T cells in the circulation. This has been managed with regular intravenous immunoglobulin infusions. Whether this leads to any long-term infectious issues and whether any other long-term toxicities exist with CAR T cell therapy are questions that are still unknown, but this is being carefully monitored and studied in previously treated patients in ongoing long-term follow-up studies.

Despite all of these advances in CAR T cell therapy, there are still unanswered questions that researchers in the field are trying to move quickly to answer. For example, the optimal CAR T cell construct and graft engineering are yet unknown, such as the best intracellular signaling costimulatory domain or generation of CAR, the ideal CD4:CD8 T cell ratio in the infused graft, or even the predominance of effector memory versus central memory cells and impact of the presence of regulatory T cells, among other factors. Identification of targets and antigen discovery in other malignancies, including not just hematologic but solid tumor malignancies as well, is another important area of ongoing study. For example, a recent study from the National Institutes of Health demonstrated the safety and efficacy of anti-BCMA CAR T cells in patients with multiple myeloma, showing impressive responses in heavily pretreated, refractory multiple myeloma [8]. CAR T cells for AML are being developed and studied as well, although the best antigen in AML is not as clear [55]. Anti-CD22 CAR T cells for ALL are also being evaluated [59]. In addition to antigen discovery, the ideal duration of engraftment of the CAR T cells is also unknown and likely differs for different malignancies. Furthermore, the impact of the tumor microenvironment is likely an important factor in CAR T cell therapy, for example the presence of inhibitory factors such as programmed-death ligand 1 (PD-L1) expression, and work is ongoing to evaluate combining immune checkpoint inhibitors with CAR T cells therapy. Moreover, as mentioned, there is work ongoing to develop strategies and new CAR T cells that may overcome antigen-negative relapse. One such strategy that is being developed is dual antigen CAR T cells targeting CD19 and CD22 in ALL, as CD19-negative relapses may still express CD22 [60]. Finally, there needs to be a focus on the very important issues surrounding technical, regulatory, and financial obstacles, so that CAR T cell manufacturing and utilization can be done a wide scale, as opposed to only being available at a handful of specialized institutions. Although these questions remain unanswered, it is clear that CAR T cell therapy is going to become an essential strategy in the treatment of hematologic malignancies, and further discoveries will only enhance the efficacy and applicability of this groundbreaking therapy.

### Immune checkpoint blockade

With the understanding that malignancies can usurp immune checkpoint pathways such as cytotoxic T-lymphocyte-associated protein 4 (CTLA-4) and programmed-death 1 (PD-1) as a mechanism of immune escape, immune checkpoint blockade was developed as a therapeutic strategy that has been shown to be effective in many solid tumors such as melanoma, non-small lung cancer, renal cell cancer, and urothelial cancer [61, 62]. Compared to solid tumor malignancies, therapy with immune checkpoint inhibitors (such as inhibitors of PD-1 and its ligand PD-L1) has yet to be fully explored in regard to potential efficacy, although the growing data on efficacy in Hodgkin’s lymphoma (HL) has been exceptional. There are several observations that suggest why HL is uniquely vulnerable to PD-1/PD-L1 blockade [63]. First, HL biopsies show Reed-Sternberg cells that are typically surrounded by an extensive (but ineffective) immune infiltrate. Second, HL is characterized by genetic alterations in 9p24.1, which results in PD-L1 and PD-L2 copy gain and overexpression, with as many as 97% of newly diagnosed classical HL biopsy specimens demonstrating 9p24.1 copy gain or amplification [64, 65]. Third, Epstein-Barr virus (EBV) infection is common in HL, which also leads to PD-L1 overexpression, which is one of the mechanisms that allow viral persistence in the host [66]. Finally, increased surface expression of PD-L1 in HL tumor biopsies has been observed.

Clinical experiences have confirmed the responsiveness of these tumors to immune checkpoint blockade. A phase 1 study evaluated nivolumab (anti-PD-1 antibody approved for use in melanoma, non-small cell lung cancer, and renal cell cancer) in patients with relapsed/refractory hematologic malignancies including MM, NHL, and HL. An expansion cohort for HL patients was performed that included 23 patients with a median of five lines of prior therapy [67]. The ORR was 87% (CR rate 17%), and PD-L1 and PD-L2 expression was observed in all tumor samples that were tested. A phase 2 study of 80 HL patients with failure after autologous stem cell transplant and either relapse or failure of subsequent brentuximab therapy showed an ORR of 66% after treatment with nivolumab [68]. Based on these data, nivolumab was approved for the treatment of relapsed/refractory HL after autologous stem cell transplant and brentuximab in May 2016. Pembrolizumab, an anti-PD-1 inhibitor approved for use in melanoma, non-small lung cancer, and head and neck cancers, has also been evaluated in HL. A phase 1b study of pembrolizumab with an expansion cohort for patients with HL evaluated 31 patients, half of whom had received five or more prior lines of therapy, and showed an ORR of 65%, with more than 70% of responses lasting longer than 24 weeks [69]. Other studies with pembrolizumab are ongoing. Ongoing research also aims to determine the ideal combination and timing of these agents in HL, but evidence clearly demonstrates an impressive responsiveness of HL to immune checkpoint blockade.

Another area where immune checkpoint blockade may play an interesting role in hematologic malignancies is after stem cell transplantation, given the unique immune environment. There is evidence of increased expression of PD-1 and PD-L1 in the setting of relapsed ALL after blinatumomab and relapsed AML, suggesting a role for inhibitors in these settings [70, 71]. The pros of this clinical setting are that it is a minimal residual disease state, immune reconstitution leads to increases in lymphocytes that are targets of PD-1 inhibition, and at least in the setting of allogeneic transplant, there may be augmentation of the graft-versus-tumor effect. However, a major con in this setting is the potential for inciting or exacerbating graft-versus-host disease after allogeneic transplant.

Pidilizumab, another anti-PD-1 inhibitor, has been used in a phase 2 study of 72 patients with diffuse large B cell lymphoma after autologous stem cell transplant [72]. The 18-month PFS was 72%, including a response rate of 55% in patients who had measurable disease after transplant. Although CTLA-4 blockade has not been as extensively studied in hematologic malignancies, ipilimumab, an anti-CTLA-4 inhibitor approved for use in melanoma, was studied in a phase 1/1b study of patients with relapsed disease after allogeneic stem cell transplant [73]. Twenty-eight patients were treated, where six patients experienced immune-related adverse events including one death and four patients experienced GVHD. Among patients who received the higher dose of ipilimumab of 10 mg/kg, two had a partial response and six had decreased tumor burden. The role of these agents in the post-allogeneic stem cell transplant setting is something that will need to be studied carefully in terms of safety and efficacy.

## Conclusions

### The future

The past and present have been extremely exciting times for immunotherapy in hematologic malignancies, but the future looks quite incredible and we are moving there quickly. Several goals are already on the horizon with ongoing research in these areas. For example, there is continuing development and refinement of antigen discovery and novel immunotherapies. We are also trying to broaden the availability of novel immunotherapies beyond just highly specialized centers. In addition, we are developing experience in the management of unique complications related to novel immunotherapies and establishing practice guidelines, which will be essential with broadening use. Moreover, it will be important to refine appropriate clinical endpoints and response assessments in studying these novel agents. Finally, combining immunotherapies is an exciting area of research that will likely further enhance our ability to harness the immune system to fight hematologic malignancies. What will be the best role for some of these novel immunotherapies, especially in the context of HCT? Are these best suited to be used as a bridge to HCT, to treat post-HCT relapse, or as a treatment in cases of transplant-ineligible patients or those without a donor? Our best hypothesis is that these novel therapies will be used as a complement to HCT with all of these clinical circumstances. Depending on many factors including durability of tumor response, a small possibility exists that novel therapies may even replace HCT in the future as a curative option for some hematologic malignancies, but for now, HCT remains as an essential therapeutic option, and combining HCT with novel therapies is a clear step in our future. Perhaps someday, the visions of both E. Donnall Thomas and Paul Ehrlich can synergize, where what has been described as the “chemotherapist’s bluntest weapon” can be combined with novel immunotherapies to achieve what is truly the “magic bullet” for patients with hematologic malignancies.

## Abbreviations

ALL: Acute lymphoblastic leukemia
AML: Acute myeloid leukemia
BAFF: B cell-activating factor
BCMA: B cell maturation antigen
BiKE: Bispecific NK cell engager
BiTE: Bispecific T cell engager
CAR: Chimeric antigen receptor
CLL: Chronic lymphocytic leukemia
CR: Complete remission
CRi: CR with incomplete count recovery
CRP: C-reactive protein
CRS: Cytokine release syndrome
CTLA-4: Cytotoxic T-lymphocyte-associated protein 4
EBV: Epstein-Barr virus
FDA: United States Food and Drug Administration
FL: Follicular lymphoma
GO: Gemtuzumab ozogamicin
GVHD: Graft-versus-host disease
HCT: Allogeneic hematopoietic stem cell transplantation
HL: Hodgkin lymphoma
IO: Inotuzumab ozogamicin
MM: Multiple myeloma
MRD: Minimal residual disease
NHL: Non-Hodgkin lymphoma
ORR: Overall response rate
OS: Overall survival
PD-1: Programmed-death 1
PD-L1: Programmed-death ligand 1
PFS: Progression-free survival
Ph: Philadelphia chromosome
